# Cribriform Adenocarcinoma of the Nasal Cavity Harboring a Novel *NAP1L1::PRKD1* Fusion, Expanding the Molecular Landscape of Minor Salivary Gland Tumors

**DOI:** 10.1155/crip/9431983

**Published:** 2025-11-27

**Authors:** Ernesto Martinez Duarte, Ross Germani, Charles Nicholson, Amy Baruch, Trisha Shattuck

**Affiliations:** ^1^Carolinas Pathology Group, Atrium Health System, Charlotte, North Carolina, USA; ^2^Bon Secours St Francis Hospital, Charleston, South Carolina, USA

## Abstract

Cribriform adenocarcinoma of the salivary glands (CASG) is a rare tumor of minor salivary glands, predominantly of the oral cavity, characterized by distinct morphologic and immunophenotypic features. In this article, we describe a unique case of CASG arising in the sinonasal cavity of a 49-year-old female, with a novel *NAP1L1::PRKD1* fusion, expanding the molecular complexities of salivary gland neoplasms. This neoplasm showed typical morphology with nests of tumor cells with cribriform and papillary architecture and a classic immunohistochemical profile with tumor cells positive for S100 and p63 while negative for p40. Molecular studies showed a *NAP1L1::PRKD1* fusion, which has not been previously detected in cribriform adenocarcinoma.

## 1. Introduction

Cribriform adenocarcinoma of the salivary glands (CASG) is a rare tumor arising from minor salivary glands of the upper aerodigestive tract [1–3]. Although controversial, this tumor is still generally considered a separate neoplasm from polymorphous adenocarcinoma (PmA) due to its unique morphologic characteristics as well as a higher risk of regional metastatic disease, though some authors consider it within the morphologic spectrum of PmA [4, 5]. CASG is currently included in the PmA heading in the fifth edition of the WHO Classification of Head and Neck Tumors [6]. These tumors can be locally aggressive and have the propensity to invade nerves, requiring careful examination of the surgical specimen [1, 7, 8].

Given their rarity, molecular characterization is limited, with studies showing few alterations that could explain their tumorigenesis and behavior [9]. We report a novel fusion in a sinonasal CAC that could provide further insights into the molecular spectrum of these tumors.

## 2. Case Presentation

A 49-year-old South Asian female presented with nasal obstruction, congestion, and epistaxis for approximately 1 year. She also complained of crusting scabs building up in the nostrils. Her symptoms were followed by mouth breathing with congestion and rhinorrhea, all worse on the right side. She has no history of trauma or prior surgical procedures on her nasal area. The patient is a never smoker and denies secondhand smoke exposure.

On a clinical exam, her nasal mucosa was red and inflamed with a deviated nasal septum. The inferior turbinate was obstructed by a mass on the right side.

A CT scan showed a right nasal mass measuring 2.6 × 1.4 cm associated with mild erosive changes of the right nasal bone. The PET scan showed that this mass was hypermetabolic with no evidence of metastatic disease.

**Figure 1:**
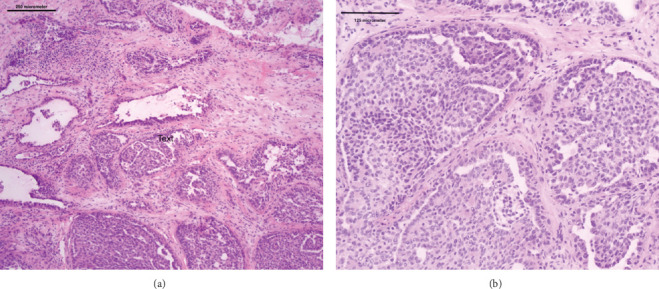
Cribriform architecture with areas showing papillary tufts. (a) 10x. (b) 20x.

An excisional biopsy was performed. The surgeon encountered a densely adherent mass on the inferior turbinate, which was resected and sent to pathology. Microscopically, the lesion showed an infiltrative growth pattern composed of nests of tumor cells forming cribriform and papillary structures separated by thick fibrous septae (Figure 1). The nuclei were ovoid and pale with clear vesicular chromatin and a mitotic count of 2 mitotic figures/mm^2^.

**Figure 2:**
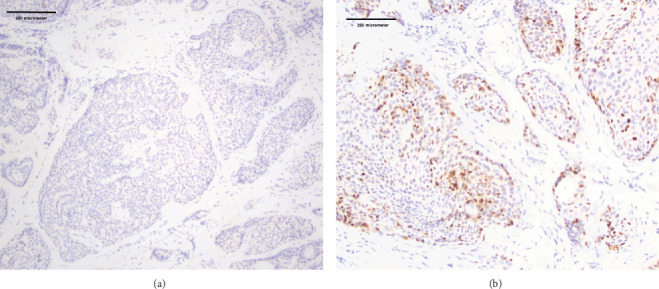
(a) Negative p40 by immunohistochemistry, while there is (b) focal p63 expression. (a, b) 10x.

By immunohistochemistry, the tumor cells were positive for cytokeratins (CK-AE1/3 and CAM5.2), S100, and p63 while negative for p40, TTF1, and GATA3 (Figure 2).

Because of the unusual location, molecular studies were requested and showed a *NAP1L1::PRKD1* fusion. This alteration was in frame and resulted in fusions of Exons 1–14 of the NAP1L1 gene to Exons 12–18 of the PRKD1 gene, leaving the protein kinase domain of the PRKD1 gene intact.

The postsurgical course was uneventful with the patient healing appropriately after definitive resection with no evidence of involvement of the lachrymal system or residual tumor.

## 3. Discussion

CASG was first described as a rare subtype of PmA predominantly arising in the tongue [10] in 1999, with cases later reported arising from the soft palate, retromolar buccal mucosa, tonsils, and lips, and rare cases reported arising from major salivary glands [5, 11–13].

To our knowledge, this tumor has not been reported arising from the sinonasal cavity which promoted additional workup. Cribriform adenocarcinoma exhibits slow growth and indolent behavior despite its avidity for nerves [13, 14]. It affects a wide age range although it tends to be more common in the sixth and seventh decades [14] with a female to male ratio of 1:2 [1, 2, 14]. The tumor tends to present with an infiltrative, multinodular growth pattern with a solid, cribriform, papillary, and microcystic architecture, separated by fibrous septae [5, 10, 11, 13]. In the background areas, conventional PmA morphology can be identified [5, 13].

CAGS and PmA are typically positive for CK, S100, SOX10, and p63 with a strikingly negative expression of p40 [15–17].

Molecular studies for the diagnosis are rarely used since the typical morphologic and immunophenotypic features of these lesions are sufficient to make the diagnosis. We performed molecular studies given the unusual location and limited sample, to give the patient the most accurate diagnosis [13]. Few unique and rare mutations have been associated with PmA, but to our knowledge, this is the first identified NAP1L1::PRKD1 fusion not only reported in a cribriform adenocarcinoma but also in one arising in the nasal cavity [18, 19].

Cribriform adenocarcinoma constitutes a challenging diagnosis even in the oral cavity where it is more commonly seen, and the differential diagnosis includes pleomorphic adenoma, adenoid cystic carcinoma, and even rare cases of metastatic disease, such as papillary thyroid carcinoma [10, 20, 21].

The current treatment for cribriform adenocarcinoma is complete surgical resection with clear resection margins. Given the avidity for nerves, recurrence can be seen and close follow-up is recommended after surgery. Studies have shown an approximately 17% rate of recurrence after follow-up [2, 22]. The role of neoadjuvant radiotherapy in this entity is still unclear, and we recommend for our patient complete surgical resection with clear margins and close postsurgery follow-up.

Interestingly, in our case, despite evidence of local advanced disease on imaging studies, there was no histologic evidence of perineural invasion or evidence of distant metastasis.

## 4. Conclusion

Although this specific alteration has not been described in the scientific literature other similar recurrent translocations in the PRKD1-3 gene family have been reported as pathogenic in cribriform adenocarcinoma and PmA. While CAGS has a classic morphology and immunohistochemical profile, molecular studies may be helpful in confirming this diagnosis when presenting in an unusual location outside the oral cavity.

## Data Availability

The data that support the findings of this study are available on request from the corresponding author. The data are not publicly available due to privacy or ethical restrictions.
